# Prevalence of Insomnia, Nightmares and Obstructive Sleep Apnea Among Medical Students: A Cross‐Sectional Study

**DOI:** 10.1002/hsr2.72402

**Published:** 2026-04-21

**Authors:** Mohamad Ala Rashi, Khaled Alkhateeb, Hamza Altabbaa, Ali Hsamo, Yahia Almouie, Mohamad Omar Harbeh, Maysoun Kudsi, Data collaborators, Sally jabbour, Ghadeer Eskandar Jawish, Sedra Sahly, Maya Ghanem, Nour Zrba

**Affiliations:** ^1^ Faculty of Medicine Damascus University Damascus Syria; ^2^ Faculty of Medicine Tartous University Tartous Syria; ^3^ Faculty Of Medicine Aleppo University Aleppo Syria; ^4^ Rheumatology Department Damascus University Damascus Syria

**Keywords:** academic performance, insomnia, medical students, nightmares, obstructive sleep apnea

## Abstract

**Background and Aims:**

This study investigates the prevalence of insomnia, nightmares, and obstructive sleep apnea among medical students and their correlation with several variables.

**Methods:**

We gathered data from six medical colleges across Syrian universities, focusing on second to sixth‐year human medicine students. The questionnaire, based on reliable international tools like the Berlin Questionnaire and Insomnia Severity Index, was distributed electronically and in print. We used the chi‐square test to analyze disease prevalence associations.

**Results:**

The sample comprised 1237 medical students with a mean age of 20.74 years (SD = 1.809), including 635 males (51.3%) and 602 females (48.7%). Insomnia was found in 46.9% of participants, while 15.8% reported nightmares, and 4.8% were at high risk for obstructive sleep apnea. Insomnia significantly affected academic performance, with 8.7% of students with GPAs between 60 and 70% experiencing severe insomnia, compared to just 1.6% of those with GPAs between 91 and 100% (*p* = 0.012). However, nightmares and obstructive sleep apnea did not significantly impact academic performance. A family history of insomnia and nightmares was linked to a higher likelihood of experiencing them (*p* < 0.05). The study found no strong association between obstructive sleep apnea and other variables, except regarding sleep duration; 40.7% of high‐risk individuals slept less than 6 h versus 24.4% of low‐risk individuals (*p* = 0.019).

**Conclusion:**

Sleep disorders are common among medical students and significantly affect their quality of life and health. Prioritizing the diagnosis of these disorders is essential, and future research should explore genetic factors related to their prevalence.

## 1 Background

Sleep constitutes a fundamental physiological necessity, marked by suppressed sensory perception, altered consciousness, and reduced voluntary muscular activity [1]. Electrophysiological analyses reveal two distinct sleep phases in most mammalian species, particularly humans: Non‐Rapid Eye Movement (NREM) sleep and Rapid Eye Movement (REM) sleep [2].

The global prevalence of sleep disorders ranges from 22% to 65% among general populations [3], with United States data showing rates of 20–41.7% during 2011. Subsequent epidemiological surveys indicate a 40.4% escalation in prevalence between 2013 and 2016 [4]. Sleep duration and restorative quality critically influence neurocognitive functioning and physiological homeostasis, necessitating their integration into therapeutic interventions [5].

Scholastic achievement patterns in pediatric and adolescent populations demonstrate significant associations with lifestyle patterns and behavioral habits. Enhanced academic outcomes correlate strongly with nutritional adequacy, regular exercise regimens, and age‐appropriate sleep duration [6]. Contemporary studies document rising sleep disorder incidence among university students globally, particularly linked to escalating academic demands and resultant stress [6]. Medical students exhibit heightened vulnerability to sleep pathologies, with multiple sleep disorders demonstrating correlations with diminished academic achievement [7]. Numerous investigations have been conducted to quantify sleep disturbance prevalence in collegiate populations [8]. Common diagnostic entities include insomnia syndromes, restless legs syndrome (RLS), circadian rhythm disruptions, affective disorders, narcolepsy, and obstructive sleep apnea (OSA) [8]. Current evidence suggests substantial proportions of university students harbor undiagnosed sleep impairments, with individuals exhibiting sleep‐related disturbances facing elevated risks of academic underperformance [9].

The societal burden of insomnia exceeds that associated with other medical and psychiatric conditions such as arthritis, depression, and hypertension. Office visit diagnoses for insomnia in the United States surged from 800,000 in 1993 to 9.4 million by 2015, reflecting an 11‐fold increase over this period. Insomnia may manifest as a primary disorder, a secondary symptom of other sleep pathologies, or a comorbid condition alongside other sleep disturbances [10]. Recent epidemiological studies report a prevalence of insomnia ranging from 9.5% to 27% among college student populations [11]. Furthermore, insomnia symptoms have been identified as significant contributors to academic performance deficits and mental health challenges in this demographic [12].

Obstructive sleep apnea (OSA) is characterized by recurrent episodes of upper airway obstruction during sleep, accompanied by arousal with or without oxygen desaturation [13]. Obstructive sleep apnea syndrome (OSAS) represents a significant public health concern, profoundly impacting life expectancy and quality of life in affected populations [14]. Epidemiological estimates indicate a prevalence of 3% in females and 10% in males aged 30–49 years, rising to 9% in females and 17% in males aged 50–70 years; approximately 24 million undiagnosed cases are reported in the United States [15]. Recent data suggest that 19% of students exhibit high‐risk indicators for OSA, with 26.4% reporting excessive daytime sleepiness [16]. The clinical ramifications of OSA extend beyond somatic health, as pediatric cases demonstrate frequent associations with learning impairments and diminished academic performance [17]. Academic underachievement in OSA may arise from hypoxemia and sympathetic‐cortical activation, which disrupt memory consolidation processes [17].

Nightmares are defined as vivid, disturbing dreams provoking awakenings, typically featuring repetitive themes of intense negative affect. When nightmares induce functional impairment in occupational, social, or psychological domains, they meet diagnostic criteria for Nightmare Disorder under parasomnia classifications. Population studies report monthly nightmare prevalence rates of 13–45%, with weekly occurrences affecting 2–6% of individuals. Etiologically, nightmares may manifest idiopathically or correlate with psychiatric comorbidities, including posttraumatic stress disorder (PTSD), anxiety disorders, substance use disorders, and schizophrenia‐spectrum conditions [18]. Students in conflict zones constitute a high‐risk demographic for recurrent posttraumatic nightmares, warranting targeted screening and therapeutic interventions [19]. Research on Chinese adolescent cohorts reveals significant associations between nightmare frequency and cognitive deficits, mediated predominantly by comorbid insomnia, depressive symptoms, and anxiety [20]. Furthermore, nightmares exhibit longitudinal associations with anxiety escalation and subsequent depression in adolescents, demonstrating persistent effects across developmental stages [21].

## 2 Patients and Methods

### 2.1 Study Design and Sample Size Calculation

This is a cross‐sectional study without a control group. From October 2024 to January 2025, we studied 1237 participants. The sample size was initially calculated to be 1014 participants with a confidence interval of 95%, a margin of error 3%, and a predictive value of 0.05, calculated by (http://datatab, net/tutorial/sample‐size). No previous study was available in Syria. This study was approved by the ethics committee of Damascus University (approval no: MD‐261124‐359, 2024). All patients were fully informed and agreed to the concept and aim of this study.

### 2.2 Participants

1237 participants were enrolled in our survey.

### 2.3 Exclusion Criteria

The inclusion criteria for the sample included students from second through sixth year in the six medical colleges mentioned who agreed to participate in the survey; exclusion criteria included preparatory medical students, students who refused participation, and cases with incomplete responses.

## 3 Methods

We collected data from six medical colleges across six universities in the Syrian Arab Republic, which are affiliated with the Ministry of Higher Education and Scientific Research. These universities include Damascus University, Aleppo University, Homs University, Tartous University, Euphrates University, and Lattakia University. The medical education system in all these colleges consists of six academic years; the first year is a preparatory year for medical colleges, and students in this year are not only students of human medicine but also dental and pharmacy students. Therefore, we did not include first‐year students in our study; data collection was limited to students from the second year through the sixth year, who are considered in our country as human medicine students.

The grading system in these universities is based on each subject being scored out of 100 points, distributed as 70 or 80 points for the theoretical part and 20 or 30 points for the practical part. A student fails if their overall grade for a subject does not reach 60 out of 100. Accordingly, the student's average is calculated for each year.

The questionnaire was developed based on several reliable international questionnaires, such as the Berlin Questionnaire to assess the risk of obstructive sleep apnea syndrome (OSAS), the Insomnia Severity Index to diagnose insomnia, and questionnaires on nightmares and disturbing dreams to diagnose nightmares. Most questions were multiple‐choice and divided into four main sections as follows:

The first section included informed consent and confirmation that all information provided would be kept confidential and used solely for scientific research purposes. It also collected some personal information such as weight, age, height, academic year, number of sleeping hours, university affiliation, and family history of three specific conditions: insomnia, obstructive sleep apnea syndrome (OSAS), and nightmares.

The second section contained questions related to obstructive sleep apnea (OSA). The first category included questions like: 1‐ Do you snore? 2‐ How would you describe this snoring? 3‐ How many times do you experience snoring? 4‐ Has your snoring disturbed others before? 5‐ Has anyone noticed that you stop breathing during sleep? The second category included questions such as: 6‐ How often do you feel tired after waking up? 7‐ During the day, do you feel tired or not at your best? 8‐ Dozing off while driving? 9‐ If yes, how many times has this happened? The third category asked: 10‐ Do you suffer from high blood pressure?

The points are calculated based on “Yes” or “No” questions, with one point awarded for a “Yes” answer. For multiple‐choice questions, two points are given for the answers that correspond to the highest severity of sleep apnea. The first and second categories are considered high risk if the individual scores two or more points. As for the third category (obesity and blood pressure), a person is considered high risk if high blood pressure is detected or if the body mass index (BMI) exceeds 30 kg/m².

Thirdly, there is a section of questions about insomnia, which includes: (1) Do you find it difficult to fall asleep during the past 2 weeks? (2) Do you find it difficult to stay asleep during the past 2 weeks? (3) Do you experience early awakening during the past 2 weeks? (4) How satisfied are you with your sleep? (5) Have others noticed the impact of insomnia on you? (6) How anxious are you about your insomnia? (7) How does insomnia affect your daily functioning?

The points are calculated based on the interpretation of the scale results: an overall score from 0–7 indicates “no significant clinical insomnia,” 8–14 means “subthreshold insomnia,” 15–21 represents “clinical insomnia (moderate severity) “and 22–28 signifies “clinical insomnia (severe).”

Fourth, the nightmare questions section, which includes (1) How many times do you experience nightmares? (2) How many nights per week do you suffer from nightmares? (3) The number of nightmares in 1 week? (4) Since when have you been experiencing nightmares? (5) Do you wake up from sleep because of nightmares? (6) How do you evaluate the problem of nightmares and their impact? (7) How do you evaluate the severity of the nightmares and their impact? Points are calculated by: adding the number of nights per week (0–7) + number of nightmares per week + question 3 score (0–4) + question 4 score (0–6) + question 5 score (0–6). “Note: The maximum number of nightmares per week is 14, so the scale ranges from 0 to 14.” Usually, if the result exceeds 10, it indicates a nightmare disorder.

The tool's reliability was reported previously; the internal consistency values were good for this instrument [22, 23, 24].

The reliability test for the questionnaire was conducted on a sample of 100 participants, yielding the following Cronbach's alpha values: insomnia 0.82, nightmares 0.76, and obstructive sleep apnea 0.7.

After developing this questionnaire, we created an electronic version and published it on social media groups dedicated to medical students at the mentioned universities. We also printed paper copies and distributed them among students at the university who did not complete it electronically, remaining nearby to assist with any issues.

## 4 Statistical Analysis

We used IBM SPSS statistical software for data analysis. Appropriate statistical tests were employed to clarify relationships between variables—for example, using Chi‐square tests [25] with a confidence interval of 95% and a predictive value of 0.05 to examine associations between the prevalence of three diseases and academic achievement among students, as well as relationships between disease prevalence and demographic variables.

## 5 Results

The study sample consisted of 1237 medical students, with a mean age of 20.74 years (SD = 1.809), ranging from 18 to 39 years. Among them, 635 were males (51.3%) and 602 were females (48.7%). Most participants were from Damascus University (58.6%), followed by Lattakia (11.2%), Aleppo (9.5%), Homs (8.2%), and Tartous (7.8%), with the fewest from Hama University (2.8%) and other institutions (1.9%). The majority were second‐year students (38.6%), followed by third‐year (19.8%), fourth and sixth‐year (14.6% each), and fifth‐year (12.3%). Regarding GPA, more than half of the students had grades between 81 and 90 (55.9%), followed by 71–80 (24.6%), 91–100 (17.6%), and 60–70 (1.9%). Approximately 48.5% of the students did not experience any of the mentioned disorders, while 36.6% experienced one, 13.7% experienced two, and 1.1% experienced all three studied disorders. The Table 1 and Table 2 show that the previous information in detail.

**Table 1:** Demographic information of students participating in the questionnaire.

		*N*	%
Gender	Male	635	51.3
	Female	602	48.7
University	Damascus	725	58.6
	Lattakia	138	11.2
	tartous	97	7.8
	Aleppo	117	9.5
	Homs	101	8.2
	Hama	35	2.8
	other	24	1.9
Academic year	2	478	38.6
	3	245	19.8
	4	181	14.6
	5	152	12.3
	6	181	14.6
University GPA	60–70	23	1.9
	71–80	304	24.6
	81–90	692	55.9
	91–100	218	17.6
Sleep hours	6 ≥	312	25.2
	6 <	925	74.8
Family history of insomnia	yes	501	40.5
	no	736	59.5
Family history of nightmares	yes	286	23.1
	no	951	76.9
Family history of OSA	yes	152	12.3
	no	1085	87.7

**Table 2:** Prevalence of sleep disorders among medical students.

		*N*	%
Insomnia	No clinically significant insomnia	656	53
	Subthreshold insomnia	432	34.9
	Moderate insomnia	129	10.4
	Severe insomnia	20	1.6
nightmares	yes	195	15.8
	no	1042	84.2
OSA	High risk	59	4.8
	Low risk	1178	95.2

Insomnia was prevalent among 46.9% of students, with 34.9% experiencing subthreshold insomnia, 10.4% moderate, and 2.2% severe insomnia. No significant gender differences were found in insomnia severity. A significant relationship existed between insomnia severity and sleep duration, with about 80% of those with severe insomnia sleeping less than 6 h. Insomnia severity increased with academic year (*p* > 0.001), with higher rates in later years: Second year (0.8%), Third year (0.8%), Fourth year (2.2%), Fifth year (2.6%), and Sixth year (3.3%). Aleppo University students had the highest rates of moderate insomnia (13.7%), while those with GPAs between 60 and 70 exhibited the highest rates of both moderate (13%) and severe insomnia (8.7%). Additionally, students with a family history of insomnia showed higher rates of moderate (14.2%) and severe insomnia (2.2%) compared to those without such a history (*p* < 0.05). Table 3 presents the significant relationships between insomnia and various studied variables.

**Table 3:** The relationship between insomnia and various variables using the chi‐square test.

		insomnia				*p* value
		No clinically significant insomnia	Subthreshold insomnia	Moderate insomnia	Severe insomnia	
Gender	male	55.9%	33.9%	8.8%	1.4%	0.11
	female	50%	36%	12.1%	1.8%	
Academic year	2	45.2%	42.3%	11.7%	0.8%	0.0003
	3	60.4%	28.6%	10.2%	0.8%	
	4	53.6%	31.5%	12.7%	2.2%	
	5	59.9%	28.3%	9.2%	2.6%	
	6	57.5%	33.1%	6.1%	3.3%	
University	Damascus	55.2%	33.2%	9.9%	1.7%	0.029
	lattakia	45.7%	35.5%	16.7%	2.2%	
	Aleppo	36.8%	47.9%	13.7%	1.7%	
	Homs	52.5%	37.6%	7.9%	2%	
	Tartous	63.9%	27.8%	7.2%	1%	
	Hama	65.7%	31.4%	2.9%	0%	
	other	50%	41.7%	8.3%	0%	
University GPA	60–70	69.6%	8.7%	13%	8.7%	0.012
	71–80	48%	37.8%	11.8%	2.3%	
	81–90	54.5%	33.5%	10.7%	1.3%	
	91–100	53%	34.9%	10.4%	1.6%	
Family history of inomnia	yes	45.3%	38.3%	14.2%	2.2%	0.000013
	no	58.3%	32.6%	7.9%	1.2%	

Nightmares were reported by 15.8% of participants, ranking second in prevalence. A significant gender difference was observed (*p* > 0.001), with females (20.3%) experiencing nightmares more frequently than males (11.5%). Students with a family history of nightmares had a higher incidence (30.1%) compared to those without (11.5%), indicating family history as a strong risk factor (*p* = 0). Lower‐year students, particularly second‐year students (20.1%), reported more nightmares, while only 10.5% of sixth‐year students experienced them, suggesting early academic years influence nightmare occurrence. No significant associations were found between nightmares and GPA or university. Table 4 presents the significant relationships between nightmares and various studied variables.

**Table 4:** The relationship between nightmares and various variables using the chi‐square test.

		Nightmares		*p* value
		yes	No	
Gender	male	11.5%	88.5%	0.000016
	female	20.3	79.7%	
Academic year	2	20.1%	79.9%	0.02
	3	17.1%	82.9%	
	4	13.8%	86.2%	
	5	8.6%	91.4%	
	6	10.5%	89.5%	
University	Damascus	14.1%	85.9%	0.373
	lattakia	16.7%	83.3%	
	Aleppo	22.2%	77.8%	
	Homs	17.8%	82.2%	
	Tartous	18.65	81.4%	
	Hama	14.3%	85.7%	
	other	12.5%	87.5%	
University GPA	60–70	13%	87%	0.919
	71–80	16.1%	83.9%	
	81– 90	15.3%	84.7%	
	91–100	17%	83%	
Family history of nightmares	yes	30.1%	69.9%	0.00000
	no	11.5%	88.5%	

The study found that 4.8% of students are at high risk for obstructive sleep apnea (OSA), while 95.2% are at low risk. The highest OSA risk rates were observed among Aleppo University students (6.8%), those with a GPA of 71–80 (6.6%), and second‐year students (6.3%). However, these differences were not statistically significant, nor were there significant associations between OSA and gender, academic year, university, GPA, or family history. Notably, 40.7% of high‐risk individuals reported sleeping 6 h or less, compared to 24.4% of low‐risk individuals who slept less than 6 h, suggesting that higher OSA risk may correlate with shorter sleep duration. Table 5 presents the significant relationships between OSA and various studied variables, while Table 6 shows the effect of different factors on the number of hours of sleep.

**Table 5:** The relationship between OSA and various variables using the chi‐square test.

		OSA		*p* value
		High risk	Low risk	
Gender	male	5.5%	94.5%	0.13
	female	4%	96%	
Academic year	2	6.3%	93.7%	0.321
	3	3.3%	96.7%	
	4	5%	95%	
	5	3.9%	96.1%	
	6	4.8%	95.2%	
University	Damascus	5.7%	94.3%	0.18
	lattakia	4.3%	95.7%	
	Aleppo	6.8%	93.2%	
	Homs	2%	98%	
	Tartous	1%	99%	
	Hama	0%	100%	
	other	0%	100%	
University GPA	60–70	4.3%	95.7%	0.390
	71– 80	6.6%	93.4%	
	81– 90	4%	96%	
	91–100	4.6%	95.4%	
Family history of nightmares	yes	4.6%	95.4%	0.294
	no	5.9%	94.1%	

**Table 6:** The impact of different variables on the number of hours of sleep among medical students.

		Sleep hours		*p* value
		6 ≥	6 <	
Gender	male	25.7%	74.3%	0.357
	female	24.8%	75.2%	
Academic year	2	25.9%	74.1%	0.160
	3	24.9%	75.1%	
	4	30.4%	69.6%	
	5	18.4%	81.6%	
	6	24.3%	75.7%	
University	Damascus	25%	75%	0.480
	lattakia	31.2%	68.8%	
	Aleppo	21.4%	78.6%	
	Homs	24.7%	75.3%	
	Tartous	25.8%	74.2%	
	Hama	14.3%	85.7%	
	other	33.3%	66.7%	
University GPA	60–70	30.4%	69.6%	0.732
	71– 80	26.3%	73.7%	
	81–90	24.6%	75.4%	
	91–100	25.2%	74.8%	
Insomnia	No clinically significant insomnia	19.7%	80.3%	0.0000
	Subthreshold insomnia	25.7%	74.3%	
	Moderate insomnia	43.4%	56.6%	
	Severe insomnia	80%	20%	
Nightmares	yes	30.8%	69.2%	0.129
	no	24.2%	75.8%	
OSA	High risk	40.7%	59.3%	0.019
	Low risk	24.4%	75.6%	

## 6 Discussion

This study aims to investigate the prevalence of major sleep disorders among medical students and to analyze the key associated factors at the leading Syrian universities, which collectively have approximately 20,000 students, including those in Damascus, Lattakia, Aleppo, Tartus, Homs, and Hama. The study has yielded several noteworthy findings that will be highlighted.

The results indicate that insomnia is the most prevalent sleep disorder among medical students, with 46.9% of the participants reporting some form of insomnia, including 10.4% experiencing moderate insomnia and 1.6% suffering from severe insomnia. These figures can be considered relatively high when compared to the global prevalence rates of insomnia, which range from 10% to 33% in the general population [26]. The prevalence of insomnia among medical students in this study is also higher compared to a previous study conducted in Jordan, which reported that 18.8% of female medical students and 17.8% of male medical students suffer from insomnia [7]. The highest rates of insomnia were observed among students in higher years, such as the fifth and sixth years, which may be attributed to increased stress and psychological pressure related to their academic and professional futures as they approach the completion of their studies. This contrasts with a study conducted in Saudi Arabia, which indicated that insomnia and poor sleep quality tend to occur more frequently among students in lower academic years [27]. The current study demonstrates a significant impact of insomnia on academic performance among medical students, where 8.7% of those with GPAs between 60% and 70% experience severe insomnia, compared to only 1.6% of students with GPAs between 90% and 100%. This is not surprising, as numerous studies indicate that poor sleep quality negatively affects academic performance and daily activities, including problem‐solving abilities in mathematics [28]. The family history of insomnia among relatives has a significant impact on the likelihood of their children experiencing insomnia, with 14.2% of individuals with a family history of insomnia suffering from moderate insomnia, compared to 7.9% of those without such a history.

Nightmares are the second most common sleep disorder among medical students, with 15.8% of students experiencing them, while the prevalence of nightmares is higher among nursing students, as reported in a study conducted in the United Arab Emirates, where 22.2% of students reported having nightmares [8]. Notably, nightmares tend to affect females more than males, with 20.3% of females experiencing nightmares compared to 11.5% of males. In contrast to insomnia, nightmares are more prevalent among lower‐year students, with 20.1% of second‐year students suffering from nightmares compared to 10.5% of sixth‐year students. The results did not indicate a strong correlation between the prevalence of nightmares among students and their academic performance (*p* = 0.919), although numerous studies highlight the significant contribution of nightmares to the prevalence of insomnia and other psychological disorders among adolescents [21]. The family history of nightmare occurrence has a significant impact on its prevalence among medical students, as 30.1% of those with relatives who suffer from nightmares also report experiencing them. This observation may prompt serious consideration of the genetic basis for this disorder.

Obstructive sleep apnea (OSA) has finally emerged as a prevalent condition among sleep disorders in medical students. Research findings indicate that 4.8% of medical students are at high risk for OSA, as assessed by the Berlin Questionnaire. This prevalence is nearly comparable to the 4% prevalence of OSA reported in a study conducted in the Southern United States, which included 1,845 college students using the Sleep 50 questionnaire [9].

This study found no statistically significant association between OSA and several studied variables, possibly due to the low number of recorded cases, except for its impact on sleep duration. Specifically, 40.7% of individuals at high risk for OSA reported an average sleep duration of less than 6 h, compared to 24.4% of those at low risk.

Numerous studies have noted that patients with OSA experience difficulties with concentration and memory [29]; in addition, Recent research has highlighted the significant association between obstructive sleep apnea (OSA) and mental health disorders, particularly noting that the high prevalence of depression and anxiety among OSA patients can exacerbate sleep disturbances and decrease treatment adherence, while patients with OSA‐related cognitive impairment (OSA‐CI) may also experience deficits in attention, memory, executive function, emotion regulation, visuospatial ability, fine coordination, and language skills [30, 31]. However, this study did not demonstrate a significant effect on academic performance (*p* = 0.390). This finding aligns with a study conducted in Saudi Arabia involving 621 medical students [32]. Nevertheless, some studies continue to suggest that OSA may impact academic achievement among university students, despite its lower prevalence in this population [33].

There is a strong link between chronic headaches and sleep disorders, particularly headaches that occur at night or in the early morning [34]. Individuals who suffer from chronic headaches often reduce their physical activity, as exercise can temporarily intensify their pain. Those who experience frequent headaches tend to spend more time resting, lying down, or sitting, which can make them feel less tired at night. Daytime naps are also common. However, reduced activity and lack of exercise can worsen depression and anxiety, further contributing to difficulties in falling asleep and staying asleep [34]. The occurrence of headaches in individuals with obstructive sleep apnea (OSA) is reported to be moderate. Recent research reveals that the overall prevalence of headaches among OSA patients is approximately 33%, which includes 33% for morning headaches, 25% for headaches associated with sleep apnea, 19% for tension‐type headaches, and 16% for migraines. Notably, the presence of OSA does not appear to elevate the risk of developing headaches. Consequently, there is a necessity for additional investigations that explore the bidirectional relationships between sleep disorders and headache conditions [35].

### 6.1 Limitations

This study aimed to include all medical students from most Syrian provinces; however, there are several universities with fewer students than those mentioned in this research that were not included due to difficulties in accessing all areas. Additionally, this research focused solely on three of the most common sleep disorders, as the authors sought to avoid overwhelming the questionnaire used. Therefore, it did not encompass many sleep disorders that may be less common than those discussed in this study, such as Restless Legs Syndrome, sleepwalking, hypersomnia, and narcolepsy, among others.

The investigation of these disorders relied on globally recognized and reliable questionnaires, such as the Berlin Questionnaire for assessing obstructive sleep apnea, the Insomnia Severity Index for evaluating insomnia, and the Nightmare and Disturbing Dreams Questionnaire for diagnosing nightmares. However, these conditions were not diagnosed by practicing specialist physicians or through the use of more advanced techniques such as polysomnography (PSG). Additionally, the questionnaires may carry some degree of bias and are less objective than PSG. Furthermore, the questionnaires did not take into account certain factors that could influence sleep disorders, such as physical and mental illnesses, medication use, work patterns, and others.

## 7 Conclusion

Sleep disorders are prevalent among medical students, as this study indicates that 51.5% of medical students experience at least one of these three disorders. Given the significant impact these disorders have on individuals' quality of life and their physical and mental health, as well as the clear effect some of them have on academic performance, it is crucial to prioritize the diagnosis of these disorders through necessary preliminary surveys for their detection. Additionally, encouraging students to seek support and assistance from specialists and making the enhancement of sleep quality a priority in health policy for medical students is essential.

Furthermore, this study paves the way for subsequent research that investigates all sleep disorders prevalent among medical students and elucidates the key mechanisms through which these disorders may affect students' cognitive and mental capacities, as well as identifying their primary areas of impact. Since the family history of these disorders has played a role in their prevalence among medical students, it would be worthwhile for future studies to focus on the genetic factors associated with these disorders, potentially uncovering specific genes responsible for their manifestation. It may also be beneficial to explore the prevalence of these disorders among other university student populations or even high school students.

## Author Contributions

**Mohamad Ala Rashi:** investigation, validation, methodology, project administration, writing – original draft, writing – review and editing.**Khaled Alkhateeb:** data curation, writing ‐ original draft. **Hamza Altabbaa:** data curation, writing ‐ original draft. **Ali Hsamo:** data curation, writing ‐ original draft. **Yahia Almouie:** data curation, writing ‐ original draft. **Mohamad Omar Harbeh:** data curation, writing ‐ original draft. **Maysoun Kudsi:** supervision.

## Funding

The authors have nothing to report.

## Ethics Statement

the ethical approval was obtained from the Biomedical Research Ethics Committee (BMREC) at Damascus University with ID number (MD‐261124‐359).

## Consent

Informed consent was obtained from all students participating in the survey by asking them at the beginning.

## Conflicts of Interest

The authors declare no conflicts of interest.

## Transparency Statement

The lead author, Mohamad Ala Rashi, affirms that this manuscript is an honest, accurate, and transparent account of the study being reported; that no important aspects of the study have been omitted; and that any discrepancies from the study as planned (and, if relevant, registered) have been explained.

## Data Availability

The data that support the findings of this study are available from the corresponding author upon reasonable request.
